# A Self‐Degradable Conjugated Polymer for Photodynamic Therapy with Reliable Postoperative Safety

**DOI:** 10.1002/advs.202104101

**Published:** 2021-12-13

**Authors:** Hongye Huang, Wensheng Xie, Qing Wan, Liucheng Mao, Danning Hu, Hua Sun, Xiaoyong Zhang, Yen Wei

**Affiliations:** ^1^ The Key Laboratory of Bioorganic Phosphorus Chemistry & Chemical Biology (Ministry of Education) Department of Chemistry Tsinghua University Beijing 100084 China; ^2^ School of Materials Science and Engineering Nanchang Hangkong University Nanchang 330063 China; ^3^ Department of Chemistry Nanchang University Nanchang 330031 China

**Keywords:** aggregation‐induced emission, biodegradable materials, conjugated polymers, photodynamic therapy

## Abstract

As a noninvasive therapeutic technique, photodynamic therapy (PDT) has attracted numerous research interests for cancer therapy. Nevertheless, the residual photosensitizers (PSs) still produce reactive oxygen species (ROS) and damage normal cells under sunlight after PDT, which limits their practical application in clinic. Herein, the authors propose a self‐degradable type‐I PS based on conjugated polymer, which is composed of aggregation‐induced emission (AIE) and imidazole units. Due to the effective conjugated skeleton and unique AIE properties, thus‐obtained polymers can effectively generate superoxide radical (O_2_^−•^) through the type‐I process under light irradiation, which is ideal for hypoxic tumors treatment. Intriguingly, under light irradiation, O_2_^−•^ produced by the conjugated polymers can further lead to the self‐degradation of the polymer to form nontoxic micro‐molecules. It not only helps to resolve the potential phototoxicity problems of residual PSs, but also can accelerate the metabolism of the conjugated polymers to avoid the potential biotoxicity of drug accumulation. This work develops a self‐degradable type‐I PS, which can turn off the generation of ROS in time after PDT, providing a novel strategy to balance the PDT effect and postoperative safety.

## Graphical abstract

A self‐degradable type‐I photosensitizer is developed based on conjugated polymer. Conjugated polymers can effectively produce O_2_^−•^ under light irradiation, which is ideal for hypoxic tumors treatment. Meanwhile, the generated O_2_^−•^ can in return degrade the polymer to form biocompatible micro‐molecules. Significantly, this self‐degradable conjugated polymer provides a novel strategy to balance the PDT effect and postoperative safety.

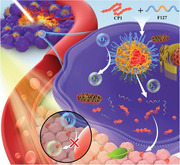

## 1 Introduction

**Figure 1:**
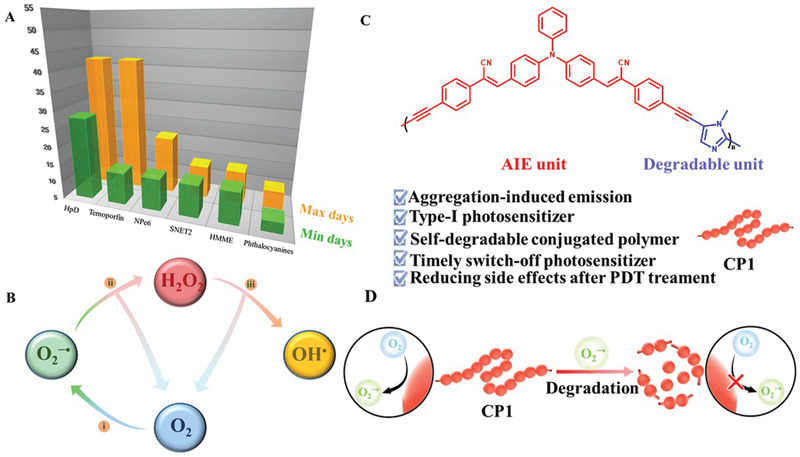
A) Therapeutic time in dark room of commercial PSs.^[^ ^7^, ^8^, ^9^, ^10^, ^11^ ^]^ B) O_2_^−•^‐driven cellular cascaded bio‐reactions for reducing the O_2_ demand of PDT. i) Type‐I photodynamic process; ii) disproportionation reaction; iii) Haber–Weiss/Fenton reaction. C) Molecular structure of self‐degradable conjugated polymer (CP1) with multifunctional performances. D) Schematic illustration of O_2_^−•^ generation from CP1 before and after degradation.

Photodynamic therapy (PDT) is a treatment that involves photosensitizers (PSs) and light irradiation to convert intracellular oxygen (O_2_) into toxic reactive oxygen species (ROS) for destroying cancer cells.^[^ ^1^, ^2^ ^]^ Nowadays, PDT has received increasing research enthusiasm, due to its high repeatability, non‐invasiveness, and non‐resistance. However, the practical application of PDT still suffers from the following two main limitations: side effects caused by residual PSs and inadequate ROS generation due to tumor hypoxia: i) because of the non‐biodegradability and non‐ideal metabolic efficiency, most of residual PSs would enter the systemic circulation and exist in normal tissue after the PDT treatment.^[^ ^3^, ^4^ ^]^ These residual PSs would sequentially produce toxic ROS when exposed under the sun‐light and then damage normal tissue to cause side effects.^[^ ^5^, ^6^ ^]^ To avoid these drawbacks, the patients are forced to stay in dark room after clinical PDT treatment (**Figure** **1**A), which in return cause inconvenience and discomfort.^[^ ^7^, ^8^, ^9^, ^10^, ^11^ ^]^ Thus, a triggered invalidation of ROS generation performance for PSs‐mediated post‐PDT treatment is essential for relieving the side effects; and ii) it is well‐known that PSs can be divided into two categories: type‐I (superoxide radical [O_2_^−•^], hydroxyl radical [OH^•^], and hydrogen peroxide [H_2_O_2_]) and type‐II (singlet oxygen [^1^O_2_]).^[^ ^12^, ^13^ ^]^ The hypoxia of tumor microenvironment (oxygen pressure <5 mm Hg) caused by the insufficient blood supply and aggressive proliferation of cancer cells will lead to inefficient ^1^O_2_ production, which greatly limits the efficiency of type‐II PSs.^[^ ^14^, ^15^, ^16^ ^]^ Fortunately, O_2_^−•^ can effectively convert into H_2_O_2_ and O_2_ through the intracellular superoxide dismutase (SOD)‐mediated disproportionation reaction (Figure 1B), which enhances the PDT efficacy even under hypoxia environment.^[^ ^17^, ^18^, ^19^ ^]^ Whereas, most commercial and reported PSs usually generated ^1^O_2_ with high oxygen dependence.^[^ ^20^ ^]^ Therefore, it is urgent to develop self‐degradable type‐I PSs that can not only turn off the production of ROS post‐treatment but also overcome decreasing PDT efficiency caused by tumor hypoxia.

Among common PSs like inorganic metallic oxide, organometallic complex, and pure organic compounds, conjugated polymers have served as attractive alternatives to other conventional PSs, due to the excellent light‐harvesting ability brought by long *π*‐conjugated backbones.^[^ ^21^, ^22^, ^23^, ^24^, ^25^, ^26^ ^]^ For example, Wang and coworkers have reported many excellent conjugated polymers for PDT.^[^ ^27^ ^]^ However, traditional conjugated polymers are always non‐biodegradable, which would prolong their metabolic time and induce undesirable side effects in vivo.^[^ ^28^, ^29^ ^]^ Meanwhile, although few novel degradable conjugated polymers have been reported, their degradation process not only needs to introduce additional complex components to build a ROS environment but also requires a long degradation time.^[^ ^30^, ^31^, ^32^, ^33^ ^]^ Thus, it is of great significance to develop the biodegradable conjugated polymers with mild degradation conditions and rapid degradation time as highly‐efficient and highly‐safe PSs for PDT.

Herein, we elaborately developed a self‐degradable conjugated polymer (CP1) with excellent O_2_^−•^ generation ability based on aggregation‐induced emission (AIE) and imidazole units to balance the PDT effect and postoperative safety (Figure 1C). Due to the extended conjugated structures and AIE units, CP1 could effectively produce O_2_^−•^ under light irradiation.^[^ ^34^, ^35^ ^]^ Meanwhile, the generated O_2_^−•^ could in return degrade the imidazole units in CP1 to form biocompatible micro‐molecules, which lost the ability to produce ROS under light irradiation (Figure 1D). Subsequently, the CP1‐based nanoparticles (CP‐NPs) are prepared for further bio‐experiment. Both in vitro and in vivo experiments demonstrated that CP‐NPs can not only inhibit the tumor growth efficiently under light irradiation, but also reduce the postoperative side effect. In general, the development of type‐I PSs based on self‐degradable conjugated polymer provides a paradigm to overcome tumor hypoxia‐induced inefficiency and side effects of PDT treatment.

## 2 Result and Discussion

As shown in Figure S2, Supporting Information, the biodegradable CP1 was synthesized through Sonogashira coupling reaction between acetylene functionalized AIE monomer (TPA‐yne) and diiodo‐N‐methyl‐imidazole. For comparison, its analogue CP2 without imidazole unit was also prepared via the same methods. On the one hand, AIE monomer not only can reduce non‐radiative decay, but also promote radiative decay and intersystem crossing (ISC) process in the aggregate state.^[^ ^36^, ^37^, ^38^, ^39^ ^]^ Furthermore, CP1 also possesses the AIE feature due to the AIE unit (Figure S3, Supporting Information). On the other hand, the imidazole unit of CP1 can provide the degradation ability of CP1 under the ROS environment.

**Figure 2:**
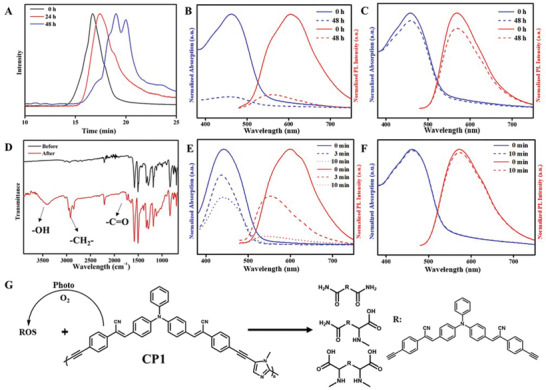
A) Normalized GPC results of CP1 after exposure to H_2_O_2_ for different time. B) Absorption and fluorescence spectra of CP1 before and after exposure to H_2_O_2_ for 48 h. C) Absorption and fluorescence spectra of CP2 before and after exposure to H_2_O_2_ for 48 h. D) FTIR spectra of CP1 before and after light irradiation for 30 min. E) Absorption and fluorescence spectra of CP1 with light irradiation (100 mW cm^−2^) for different time. F) Absorption and fluorescence spectra of CP2 with light irradiation (100 mW cm^−2^) for different time. G) Proposed mechanism for the self‐degradation of CP1. Concentrations: 20 µg mL^−1^ (CP1), 20 µg mL^−1^ (CP2), and 50 µm (H_2_O_2_).

To compare the biodegradability of CP1 and CP2 (Figure S2, Supporting Information), we treated the solution (DMF: H_2_O = 1: 99) of CP1 and CP2 (20 µg mL^−1^) with H_2_O_2_ (50 µm) for 48 h. After incubation with H_2_O_2_, the gel permeation chromatography (GPC) trace of CP1 shows that the number‐average molecular weight (Mn) of the polymer changes from 7539 to 692 (**Figure** **2**A and Table S1, Supporting Information). In contrast, the molecular weight of CP2 is almost unchanged after the treatment (Figure S4 and Table S1, Supporting Information). The GPC results provide direct evidence for the degradation of CP1 in H_2_O_2_. To further verify the degradability of CP1 and CP2 in H_2_O_2_, we also monitor their fluorescence changes upon exposure to H_2_O_2_. CP1 solution exhibits a strong red emission centered at 605 nm. Upon incubation with H_2_O_2_ for 48 h, the fluorescence of CP1 is blue shifted and almost quenched (Figure 2B, red line). The reason of blue shift is that the degradation process can break the conjugate backbones and weaken the TICT effect of CP1. Moreover, the reason of quenching is that the increasing polarity of degradation products would make their fluorescence quenching in a polar solvent (H_2_O). Furthermore, the absorption of CP1 significantly decreases upon incubation with H_2_O_2_ for 48 h, indicating that the conjugated structures are destroyed (Figure 2B, blue line). Nevertheless, the fluorescence and the absorption spectra of CP2 keep unchanged after being treated with H_2_O_2_ (Figure 2C). These results confirm that CP1 with imidazole units is easier to realize biodegradation than CP2 without the imidazole unit in the presence of biologically relevant concentrations of H_2_O_2_.

Because of the stronger intramolecular charge (ICT) state of CP1 with D–A structure, it is susceptible to generate ROS under light irradiation. Therefore, we speculate that the ROS produced by CP1 can degrade polymers, which can be called self‐degradation. Based on the previous description of imidazole degradation, we expect that the imidazole unit can be decomposed into amino acids and amides derivatives by self‐degradation (Figure 2G).^[^ ^29^, ^30^, ^40^ ^]^ To further verify this expectation, we studied the changes in the structures and properties of the conjugated polymer under light irradiation. First, 20 µg mL^−1^ CP1 and CP2 solutions (DMSO: H_2_O = 1: 99) are exposed to white light (100 mW cm^−2^) for different time. Then, the GPC data of CP1 is tested under light irradiation with different time. The results show that Mn of CP1 changes from 7000 to 1000 with the extension of irradiation time (Figure S5 and Table S2, Supporting Information), which directly confirms the self‐degradation of CP1. In contrast, the molecular weight of CP2 is unaffected after light irradiation (Figure S6 and Table S2, Supporting Information). Additionally, the FTIR analysis shows three new peaks at 2290, 3394, and 1732 cm^−1^ for CP1 after light irradiation, respectively (Figure 2D). These peaks can be attributed to the hydroxyl, methyl, and carbonyl respectively, suggesting the self‐degradation of imidazole. In addition, signals corresponding to acid amides, methine, and carboxyl groups were found in the ^1^H‐NMR spectra of the irradiated products, which originated from the degraded imidazole (Figure S7, Supporting Information). Hence, these data strongly demonstrate that light irradiation can oxidize imidazole units and degrade CP1 into various fragments containing amino acids and amides.

Except for the structural change, the properties of the conjugated polymer are also changed after self‐degradation. Figure 2E shows the fluorescence spectra (red line) and absorption spectra (blue line) of CP1 with different irradiation time. After exposure to light for 3 min, the fluorescence of CP1 is blue‐shifted, which could be explained by the decreased TICT effect due to the degradation of the conjugated structure. Furthermore, the fluorescence of CP1 is almost quenched after light irradiation for 10 min. This phenomenon is attributed to the fact that the polarity of degradation products increases, which makes their fluorescence quenching in a polar solvent (H_2_O). Moreover, the absorption peaks of CP1 gradually decrease with the increase of irradiation time, implying the degradation of conjugated backbones. On the contrary, the analogue CP2 exhibits excellent fluorescence stability under light irradiation (Figure 2F, red line). Additionally, the absorption spectra of CP2 show no obvious changes under light irradiation (Figure 2F, blue line). Taken together, all the above results further illuminate that the self‐degradation of CP1 can be induced by light irradiation.

**Figure 3:**
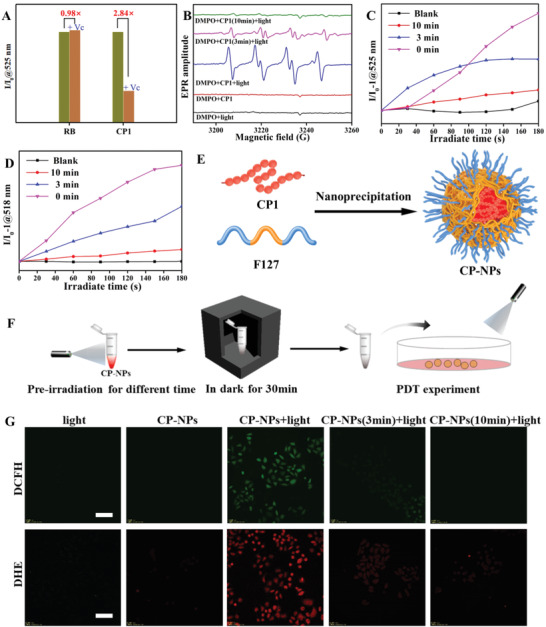
A) Relative fluorescence intensity of DCFH‐DA at 525 nm after different treatments. *I*_0_ refers to the fluorescence intensity of the no Vc group. B) EPR signals of DMPO in the presence of CP1 solution with different pre‐irradiation time. C) Plot of the relative fluorescence intensity of DCFH‐DA (50 µm) solution containing CP1 (different pre‐irradiation time) versus the irradiation time (100 mW cm^−2^). D) Plot of the relative fluorescence intensity of HPF (10 µm) solution containing CP1 (different pre‐irradiation time) versus the irradiation time (100 mW cm^−2^). E) Schematic illustration of the preparation of CN‐NPs. F) Schematic illustration of pre‐irradiation of CP‐NPs and subsequent PDT experiment. G) General ROS and O_2_^−•^ generation of CP‐NPs (different pre‐irradiation time) in HeLa cells under light irradiation (100 mW cm^−2^) by using DCFH‐DA (10 µm) and DHE (10 µm). Scale bar: 50 µm.

To reduce the potential side effects of PDT, the ROS generation ability should be invalid after the treatment. Therefore, we further evaluate the variation of ROS generation before and after the self‐degradation of the conjugated polymer. It is well known that 2′, 7′‐dichlorodi‐hydrofluorescein diacetate (DCFH‐DA) is a common green fluorescent probe for the detection of ROS including ^1^O_2_ and O_2_^−•^.^[^ ^41^ ^]^ Meanwhile, vitamin C (Vc), as a well‐known radical scavenger,^[^ ^42^ ^]^ can be used to verify whether increased DCFH‐DA signal is induced by produced O_2_^−•^. As shown in **Figure** **3**A and Figure S8A, Supporting Information, the fluorescence signal of DCFH‐DA can be observed in CP1 solution after light irradiation, implying the ROS generated by CP1. While, Vc decreases the fluorescence intensity of DCFH‐DA by 2.84 times, suggesting that the ROS generation of CP1 is the O_2_^−•^. Moreover, the fluorescence intensity is unchanged after the addition of Vc to a DCFH‐DA and RB (a commercial ^1^O_2_ PS) solution (Figure S8B, Supporting Information). Afterward, 9,10‐anthracenediyl‐bis(methylene)‐dimalonic acid (ABDA), as a commercial ^1^O_2_ probe, is further used to detect the ^1^O_2_ generation of CP1 under light irradiation. As shown in Figure S9, Supporting Information, the absorption peak at 378 nm of ABDA (blank group and CP1 group) decreased slightly with the increase of irradiation time, suggesting the low efficiency of ^1^O_2_ generation by CP1. As a comparison, the absorption peak of ABDA (RB group) decreased quickly under light irradiation (Figure S9C, Supporting Information). To further validate the O_2_^−•^ generation of CP1, the electron paramagnetic resonance (EPR) spectroscopy using 5,5‐dimethyl‐1‐pyrroline‐N‐oxide (DMPO) as the spin‐trap agent is carried out to measure the species of oxygenous radicals. Figure 3B (blue line) displays the typical EPR signal of DMPO and O_2_^−•^ additive compound after adding CP1 under light irradiation, which corresponds with previous reports.^[^ ^17^ ^]^ These results demonstrate that CP1 is the type‐I PS.

Based on the fact that the ROS generation of CP1 is O_2_^−•^, we further study the changes of ROS generation induced by CP1 before and after self‐degradation. First, CP1 is treated with self‐degradation via light irradiation for different time. Then, the DCFH‐DA is used to measure the ROS generation by CP1 with different self‐degradable time. As shown in Figure 3C, the fluorescence intensity of the blank group (pure DCFH‐DA solution) varied slightly during the light irradiation (Figure S10A, Supporting Information). After the addition of CP1 without pre‐irradiation, the fluorescence intensity of DCFH‐DA increases rapidly accompanied by the increase of light irradiation time (Figure S10B, Supporting Information). Interestingly, the fluorescence intensity of 3 min pre‐irradiation CP1 (Figure S10C, Supporting Information) is ultimately less than that of CP1 without self‐degradation. Moreover, the fluorescence intensity of 10 min pre‐irradiation CP1 (Figure S10D, Supporting Information) is close to the blank group, indicating almost no ROS generation by 10 min pre‐irradiation CP1. It should be noted that O_2_^−•^ can be transformed to OH^•^ through secondary reactions. Therefore, we further study the generation of the secondary product OH^•^, which can be specifically detected by hydroxyphenyl fluorescein (HPF). As shown in Figure 3D, the enhanced fluorescence signal is observed in the HPF solution containing CP1 without pre‐irradiation. Nevertheless, the final fluorescence intensity of 3 and 10 min pre‐irradiation CP1 is less than CP1 without pre‐irradiation. In addition, the EPR results are also in accord with this phenomenon. As displayed the Figure 3B, the EPR signal gradually decreases with the increase of self‐degradable time. In a word, these data directly highlight the decrease of O_2_^−•^ generation of CP1 induced by self‐degradation.

Considering the excellent self‐degradation performance discussed above, the in vitro performance of CP1 in cells is also explored. To endow CP1 with water solubility for biomedical applications, pluronic F127 is employed to encapsulate CP1 via the nanoprecipitation method to prepare the hydrophilic nanoparticles (CP‐NPs) (Figure 3E). The resulting nanoparticles (CP‐NPs) possess uniform spheroidal morphology based on a transmission electron microscope (TEM) image (Figure S12, Supporting Information). The average hydrodynamic diameter of CP‐NPs is 70.32 nm with a narrow size distribution (PDI = 0.194) (Figure S13, Supporting Information). Under light irradiation, CP‐NPs would be disrupted due to the degradation of CP1 (Figure S14, Supporting Information). More importantly, CP‐NPs solution possesses excellent fluorescence stability in the dark (Figure S15, Supporting Information). To evaluate the cellular uptake in vitro, cell imaging is investigated for CP‐NPs using HeLa cells via confocal laser scanning microscopy (CLSM). As indicated by CLSM images in Figure S16, Supporting Information, the blue fluorescence from cell nuclei is surrounded by homogeneous red fluorescence from the cytoplasm, demonstrating that CP‐NPs can enter cells and majorly locate at cell cytoplasm. Additionally, we also verify the self‐degradation of CP‐NPs in the cell via CLSM (Figure S17, Supporting Information). When the cells are exposed to light, the fluorescence signals from CP‐NPs gradually weaken, indicating their successful self‐degradation in the cell.

Given the O_2_^−•^ generation depending on the self‐degradable degree of CP1, we further explore photoactivity of CP‐NPs with different self‐degradable time in cells via DCFH‐DA and dihydroethidium (DHE) (a commercial O_2_^−•^ probe in the cell).^[^ ^43^ ^]^ First, CP‐NPs require self‐degradable preprocessing under light irradiation for 0, 3, and 10 min (Figure 3F). After incubating cells with a medium containing 20 µg mL^−1^ preprocessed CP‐NPs for 1 h, the cells stained with ROS or O_2_^−•^ probes are observed by CLSM (Figure 3F). When using DCFH‐DA as an indicator, the significant ROS generation of untreated CP‐NPs upon light is visualized via intracellular green fluorescence. Nevertheless, the fluorescence intensity of self‐degradable CP‐NPs is gradually quenched and accompanied by the increase of self‐degradable time, suggesting the decrease of ROS production of CP‐NPs in cells caused by their self‐degradation. As shown in Figure 3G, the cells incubated with DHE and CP‐NPs upon exposure to light emit bright red fluorescence, demonstrating the efficient O_2_^−•^ production by CP‐NPs in the cell. On the contrary, only weak DHE fluorescence signals can be noticed for pre‐irradiation CP‐NPs. All above data further indicate that CP‐NPs effectively produce the O_2_^−•^ under light irradiation in the cell, while their degradation product behaved with an inappreciable O_2_^−•^ generation ability.

**Figure 4:**
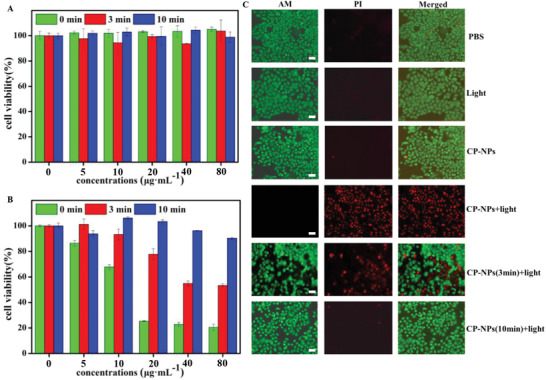
A) HeLa cells viability with CP‐NPs (different pre‐irradiation time) under dark. B) HeLa cells viability with CP‐NPs (different pre‐irradiation time) upon the white light irradiation for 3 min. C). Cell images of PI/Calcein‐AM (AM) co‐stained HeLa cells after CP‐NPs (different pre‐irradiation time) (20 µg mL^−1^) or PBS incubation under different conditions. Scale bar: 50 µm.

Given effective light‐triggered O_2_^−•^ production of CP‐NPs and the negligible O_2_^−•^ generation of their degradation product, we further explore the intracellular PDT efficiency of CP‐NPs before and after self‐degradation. Initially, the cytotoxicity of CP‐NPs is tested with HeLa cells by Cell Count Kit‐8 (CCK‐8) Assays. As shown in **Figure** **4**A, both original CP‐NPs and self‐degradable CP‐NPs show extremely low cytotoxicity. Upon exposure to light for 3 min, the untreated CP‐NPs exhibit outstanding PDT efficiency (Figure 4B). In contrast, the phototoxicity of CP‐NPs with 3 min pre‐irradiation declines significantly. More importantly, in 10 min pre‐irradiation CP‐NPs with light irradiation group, the cell viability keeps more than 90%, indicating negligible phototoxicity of self‐degradable CP‐NPs. Therefore, the untreated CP‐NPs possess great biocompatibility and excellent PDT efficiency. More importantly, CP‐NPs can “self‐degrade” and exhibit inappreciable phototoxicity after PDT.

We also employ the live/dead cell co‐staining assay to intuitively assess the PDT efficiency and safety of CP‐NPs. As shown in Figure 4C, the cells in the PBS group, light‐ group, and NPs group all showed green fluorescence, indicating that CP‐NPs have excellent biocompatibility. After being treated with CP‐NPs and irradiated for 3 min, the cells exhibit red fluorescence, indicating the effective PDT efficiency of CP‐NPs. Inversely, the vast majority of cells display green fluorescence and a small part of cells show red fluorescence in 3 min pre‐irradiation CP‐NPs with light irradiation group, demonstrating their incomplete degradation. Furthermore, no cell death is observed in 10 min pre‐irradiation CP‐NPs with light irradiation group, indicating an extremely low phototoxicity of their degradation product. Moreover, we also estimate the PDT efficiency and safety of CP‐NPs in 4T1 cells. The results of CCK‐8 Assays (Figure S18, Supporting Information) and live/dead cell co‐staining assay (Figure S19, Supporting Information) suggest the great PDT efficiency of CP‐NPs and negligible phototoxicity of their degradation product, which coincide with the results of HeLa cell. In brief, CP‐NPs not only possess excellent PDT efficiency, but can also reduce the side effects induced by residual PSs after self‐degradation, which is expected to have a high potential in PDT applications.

**Figure 5:**
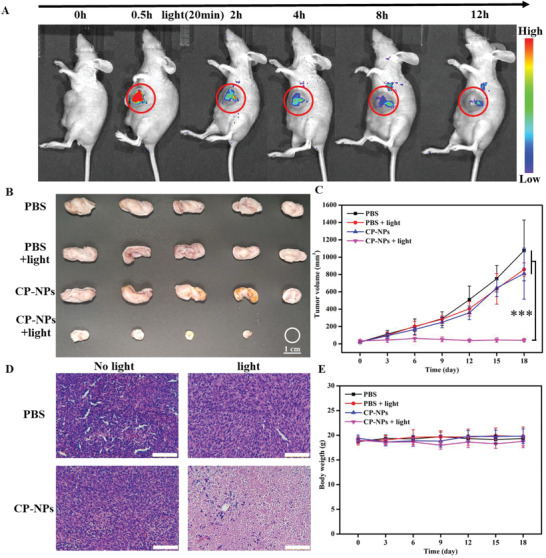
A) Fluorescence images of tumor‐bearing nude mice after intratumoral injection of CP‐NPs (200 µg mL^−1^) at different time. The tumor area is irradiated with white light (200 mW cm^−2^) for 20 min after treatment with CP‐NPs for 30 min. B) Representative photographs of the excised tumor with different treatments. Scale bars: 1 cm. C) Tumor growth curves in the different treatment groups at different times. Data were shown as mean ± SD (*n* = 5), ***p < 0.001 determined by Student's *t*‐test. D) H&E staining of tumor slices from mice after different treatments. Scale bars: 100 µm. E) Body weight of mice in the different treatment groups at different times.

Considering the outstanding in vitro PDT efficiency and inappreciable cytotoxicity, we further investigated the various performances of CP‐NPs on subcutaneous 4T1 tumor‐bearing nude mice. All animal experiments were approved by the laboratory animal research center of Tsinghua University (Animal Welfare Assurance no. F16‐00228; A5061‐01). First, we assess the self‐degradation ability of CP‐NPs in vivo through fluorescence imaging. After intratumoral injection of CP‐NPs for 12 h, the intense fluorescence signal at the tumor sites and surrounding tissues can be observed clearly, demonstrating their ideal tumor retention abilities and potential in tumor imaging (Figure S20, Supporting Information). For comparison, another group is irradiated with white light (200 mW cm^−2^) for 20 min after intratumoral injection of CP‐NPs for 30 min. As shown in **Figure** **5**A and Figure S21, Supporting Information, the fluorescence signals at the tumor sites in the light group show a sharp decline and faded away in 12 h. Furthermore, various organs and tumor tissues are obtained for imaging to verifying the residual CP‐NPs in these tissues. Thereinto, the tumors in the no light group emit bright fluorescence, but the tumors in the light group emit only weak fluorescence signals (Figures S22 and S23, Supporting Information). All these results demonstrate the successful self‐degradation of CP‐NPs in vivo.

Next, we further investigate the PDT efficiency of CP‐NPs on subcutaneous 4T1 tumor‐bearing nude mice. When the tumor volume reaches ≈100 mm^3^, the mice are randomly divided into four groups (*n* = 5): “PBS”, “PBS + Light”, “CP‐NPs”, and “CP‐NPs + Light”. The intratumoral injected dose of PBS and CP‐NPs (200 µg mL^−1^) is 25 µL/50 mm^3^ tumor and the power density of white light is 200 mW cm^−2^. As displayed in Figure 5B,C, the tumor volume in the “PBS,” “PBS + Light”, and “CP‐NPs” groups rise sharply and tumor growth cannot be inhibited. On the contrary, the tumors volume in the “CP‐NPs + Light” group is almost steady and even diminishes. For further evaluating the antitumor efficacy of CP‐NPs, the tumors of each group are collected and sliced for hematoxylin and eosin (H&E) staining. Only the “CP‐NPs + Light” group shows the obvious tissue recovery, but the other groups are closely aligned without any change (Figure 5D). More importantly, the H&E‐stained slices of the main organs show no abnormal changes (Figure S24, Supporting Information) and the weight of mice remains stable throughout the whole observation period (Figure 5E). All in all, the whole in vivo experiment and data indicate CP‐NPs not only possess excellent PDT efficiency but also can reduce the phototoxicity and side effects via the “self‐degradation” in vivo.

## 3 Conclusion

In summary, we have successfully developed a self‐degradable conjugated polymer composed of alternating AIE and imidazole units, which possess excellent PDT efficiency and reliable biosafety after PDT treatment. Due to the continuous conjugated structure and AIE unit, CP1 can effectively generate O_2_^−•^ through the type‐I process under light irradiation, which is ideal for tumor treatment. Intriguingly, the O_2_^−•^ produced by the conjugated polymers under light irradiation can lead to the self‐degradation of the polymer, accompanying the switch‐off of ROS generation. It should be noted that the degradation product of CP1 possesses extremely low phototoxicity. For further biomedical application, CP‐NPs are prepared through the assembly of CP1 and F127 to improve biocompatibility and water dispersibility. Both in vitro and in vivo experiments demonstrate that CP‐NPs can not only kill cancer cells efficiently upon light irradiation, but also avoid phototoxicity after PDT treatment via self‐degradation induced ROS generation “switch‐off”. Therefore, it is promising that this strategy provides a way to balance the therapy efficiency and postoperative safety for PDT treatment in clinic.

## Conflict of Interest

The authors declare no conflict of interest.

## Supporting information

Supporting Information

## Data Availability

Research data are not shared.
