# Supplementary material for: A Self‐Degradable Conjugated Polymer for Photodynamic Therapy with Reliable Postoperative Safety
Source: Adv Sci (Weinh). 2021 Dec 13;9(4):2104101. doi: 10.1002/advs.202104101 (PMC8811814; doi:10.1002/advs.202104101)
Supplement: Supplementary file 1 — Supporting Information [file ADVS-9-2104101-s001.pdf]

ADVANCED SCIENCE
Open Access

Supporting Information

for *Adv. Sci.*, DOI: 10.1002/advs.202104101

A Self-Degradable Conjugated Polymer for Photodynamic Therapy with Reliable Postoperative Safety

Hongye Huang, Wensheng Xie, Qing Wan, Liucheng Mao, Danning Hu, Hua Sun, Xiaoyong Zhang and Yen Wei**

Supporting Information

A Self-Degradable Conjugated Polymer for Photodynamic Therapy with Reliable Postoperative Safety

Hongye Huang, Wensheng Xie, Qing Wan, Liucheng Mao, Danning Hu, Hua Sun, Xiaoyong Zhang and Yen Wei**

H. Huang, W. Xie, L. Mao, D. Hu, H. Sun, Prof. Y. Wei
The Key Laboratory of Bioorganic Phosphorus Chemistry & Chemical Biology (Ministry of Education), Department of Chemistry, Tsinghua University, Beijing 100084, China
E-mail: weiyen@tsinghua.edu.cn

Prof. X. Zhang
Department of Chemistry, Nanchang University, Nanchang 330031, China
E-mail: zhangxiaoyong@ncu.edu.cn

Dr. Q. Wan
School of Materials Science and Engineering, Nanchang Hangkong University
Nanchang 330063, China

Experimental section

Materials and Measurements

All chemicals used in this work were purchased from commercial suppliers and used without further purification unless otherwise noted.

Gel permeation chromatography (GPC) analyses of polymers were performed using N, N-dimethyl formamide (DMF) containing 50 mM LiBr as the eluent. The GPC system was a Shimadzu LC-20AD pump system consisting of an auto-injector, a MZ-Gel SDplus 10.0 m guard column (50×8.0 mm, 10^2 Å) followed by two PLgel 5 m MIXED-D columns (300×7.5 mm), a Shimadzu SPD-20A UV/VIS detector. The system was calibrated with narrow molecular weight distribution polystyrene standards ranging from 200 to 106 g mol^{-1}. ^1H NMR and ^{13}C NMR spectra were recorded on a JEOL 400 MHz spectrometer. The FT-IR spectra were recorded in a transmission mode on a Perkin-Elmer Spectrum 100 spectrometer (Waltham, MA, USA). The mass spectra were measured using a MALDI-TOF mass spectrometer (AXIMA-PerformanceMA, Shimadzu, Japan). The size distribution of CP-NPs was measured by a Brookhaven ZetaPlus zeta-potential analyzer. UV-vis absorption spectra were recorded using UV/VIS/NIR spectrometer (Perki Elmer, Lambda 750). The fluorescence spectra were recorded using a Shimadzu RF-6000 spectrofluorometer. The Electron paramagnetic resonance (EPR) measurements were carried out on X-band EPR Spectrometer (JEOL, JES-FA200). The morphology of CP-NPs was observed by Transmission electron microscopy (TEM) (HT7700, Hitachi, Japan). The cell imaging was collected using a confocal laser scanning microscope (CLSM, Olympus FV3000, Japan). Cell viability was measured via a CCK-8 assay by a VICTOR™ X3 PerkinElmer2030 Multilabel Plate Reader.

Synthesis of TPA-CHO

Triphenylamine (500 mg, 2 mmol) was dissolved in dry DMF (10 mL). The phosphoryl chloride (20 mL) was added dropwise to the mixture at 0 °C, and the mixture was stirred for 1 h at room temperature. Then, the reaction mixture was heated to 80 °C for 8 h under the N_2 atmosphere. Finally, the reaction solution was poured into ice-cold water (20 mL) to obtain the yellow precipitate. The residue was purified by column chromatography using ethyl acetate-petroleum ether as the eluent to give TPA-CHO as a yellow solid. ^{1}H NMR (400 MHz, CHLOROFORM-D) δ 9.89 (s, 2H), 7.77 (d, J = 8.6 Hz, 4H), 7.40 (t, J = 7.8 Hz, 2H), 7.25 (d, J = 6.7 Hz, 1H), 7.18 (dd, J = 8.0, 4.4 Hz, 6H). ^{13}C NMR (101 MHz, CHLOROFORM-D) δ 190.70 (s), 152.13 (s), 145.62 (s), 131.45 (s), 130.29 (s), 127.20 (s), 126.40 (s), 122.89 (s), 77.16 (s).

Synthesis of TPA-yen

The mixture of TPA-CHO (300 mg, 1 mmol) and 4-Ethynylphenylacetonitrile (141 mg, 1 mmol) were dissolved in anhydrous methanol (30 mL) and removed into reaction bulb. Then, the piperidine (5 drops) was added and the mixture was heated to reflux for 8 h. The methanol solution was removed by rotary evaporation and the crude product was separated by column chromatography on silica gel using ethyl acetate-petroleum ether. ^{1}H NMR (400 MHz, CHLOROFORM-D) δ 7.84 (d, J = 8.8 Hz, 2H), 7.63 (d, J = 8.7 Hz, 2H), 7.55 (d, J = 8.5 Hz, 2H), 7.48 (s, 1H), 7.38 (t, J = 7.7 Hz, 1H), 7.25 – 7.14 (m, 4H), 3.19 (s, 1H). ^{13}C NMR (101 MHz, CHLOROFORM-D) δ 149.08 (s), 141.97 (s), 135.18 (s), 132.86 (s), 131.04 (s), 130.10 (s), 128.06 (s), 126.76 (s), 125.73 (s), 123.14 (s), 122.66 (s), 118.32 (s), 83.14 (s), 79.09 (s), 77.16 (s).

Synthesis of CP1

The preparation of CP1 is through Sonogashira polymerization. TPA-yen (54 mg, 0.1mmol),

2,5-Diiodo-1-methylimidazole (33 mg, 0.1mmol), $Pd(PPh_3)_2Cl_2$ (0.5 mg) and CuI (2mg) were dissolved well in ethyl acetate (5 mL). After degassing for 30 min by bubbling with N_2, triethylamine (0.5 mL) was injected to initiate the Sonogashira reaction and the mixture was kept at 75 °C for 6h. Then, the reaction mixture would precipitate a red solid, which could be separated via centrifugation. The final product was purified by washing with ethyl acetate three times.

Synthesis of CP2

The preparation of CP2 was similar to CP1.

Synthesis of CP-NPs

Briefly, CP1 (3mg) and F127 (60mg) were dissolved well in DMSO (3mL). Then, the mixture solution was dropwise added into 10 mL PBS under sonication. After sonication for 1 h, the solution was dialyzed against PBS for 3 days to remove DMSO (MWCO: 3000 Da). Finally, the concentration of CP1 encapsulated in the nanoparticles was calculated by UV/VIS/NIR spectrophotometer at 450 nm. After being purified by a 0.2 μm filter, the resultant sample CP-NPs was stored at 4 °C for further usage.

ROS generation measurement in aqueous media

2′, 7′-dichlorodi-hydrofluorescein diacetate (DCFH-DA) (1 mM in Ethanol) was treated with NaOH (1 mM in H_2O) for 30 min to obtain the DCFH. The final concentration of DCFH was 50 μM. Then, the 1 μg/mL CP1 solution (stock solution: 1mg/mL in DMSO) require a self-degradable preprocessing under the white light irradiation (100 mW/cm^2) for 0, 3 and 10 min and let stand in dark for 1 h. Afterward, CP1 with different irradiation time were added into the solution of DCFH for ROS generation measurement. The fluorescence signals were recorded

under 20 mW/cm^2 white light irradiation every 30 s. The maximal emission wavelength of the DCFH solution was at 526 nm with an excitation wavelength of 488 nm.

•OH generation measurement in aqueous media

The •OH generation measurements were conducted using hydroxyphenyl fluorescein (HPF) as the Indicator. The 1 μg/mL CP1 solution (stock solution: 1 mg/mL in DMSO) require a self-degradable preprocessing under the white light irradiation (100 mW/cm^2) for 0, 3 and 10 min and let stand in dark for 1 h. Afterward, CP1 with different pre-irradiation time were added into the PBS buffer solution containing 10 μM HPF (stock solution: 5 mM in DMF). The fluorescence signals were recorded under 20 mW/cm^2 white light irradiation every 30 s. The maximal emission wavelength of HPF solution was at 514 nm with an excitation wavelength of 480 nm.

$^1O_2$ generation measurement in aqueous media

The $^1O_2$ generation measurements were conducted using and 9,10-anthracenediyl-bis(methylene) dimalonic acid (ABDA) as the indicator. The 1 μg/mL CP1 solution (stock solution: 1mg/mL in DMSO) require a self-degradable preprocessing under the white light irradiation (100 mW/cm^2) for 0, 3 and 10 min and let stand in dark for 1 h. Afterward, CP1 with different pre-irradiation time were added into PBS buffer solution containing 20 μM ABDA (stock solution: 10 mM in DMSO). The absorption spectra were recorded under 20 mW/cm^2 white light irradiation every 30 s. The absorbance decline relative to the initial value at 380 nm was recorded to indicate the decomposition rates of ABDA ($^1O_2$ generation rate).

Cellular study

Cytotoxicity evaluation of CP-NPs

The cell viability values of CP-NPs were determined via a counting kit-8 (CCK-8) assay using HeLa and 4T1 cells. Cells were planted in 96-well plates with a density of 10000 cells per well and cultured one day. Prepared CP-NPs solution requires a self-degradable preprocessing under the white light irradiation (100 mW/cm^2) for 0, 3, and 10 min and let stand in dark for 3 h. Then, the preprocessed CP-NPs with a series of concentrations from 5 to 80 μg/mL were added and incubated at 37 °C for 24 h in dark. After that, 10 μL of CCK-8 dyes and 100 μL of Dulbecco's Modified Eagle's Medium (DMEM) cell culture media were added to each well and incubated for 2 h at 37 °C. The absorbance of sample and control wells at 450 nm was recorded via a microplate reader. Cell viability was calculated based on the absorbance data. Three replicate wells were used for each control and test concentrations per microplate, and the experiment was repeated three times.

Confocal microscopic imaging

Hela cells were seeded in a confocal imaging dish maintained at 37 °C under a humidified condition of 5% CO_2. Until the cell density reached 60-70% confluence, HeLa cells were first incubated with a culture medium containing 20 μg/mL CP-NPs for 0 and 2 h. Then, the old culture medium was removed and a fresh culture medium containing 1 μg/mL Hoechst33258 was added. After 30 min of culture, cell images were conducted with CLSM. For CP-NPs, the excitation wavelength was 450 nm and the emission filter was 580-620 nm. For Hoechst33258, the excitation wavelength was 405nm and the emission filter was 430-480 nm.

General ROS detection in vitro

HeLa cells were incubated in a confocal imaging dish until the cell density reached 60-70% confluence. Prepared CP-NPs solution requires a self-degradable preprocessing under the white

light irradiation (100 mW/cm^2) for 0, 3, and 10 min and let stand in dark for 1 h. Then, HeLa cells were cultured with a medium containing 20 μg/mL preprocessed CP-NPs solution for 4 h and treated with a medium containing 10 μM DCFH-DA solution for 30 min at 37 °C, followed by white light irradiation at 20 mW/cm^2 for 3 min. Afterward, the signal intensity was recorded by CLSM at excitation of 488 nm laser. Light alone and CP-NPs alone were stained like the above procedures. The emission filter was 495-550 nm.

$O_2^{-\bullet}$ detection in vitro

HeLa cells were incubated in a confocal imaging dish until the cell density reached 60-70% confluence. Prepared CP-NPs solution requires a self-degradable preprocessing under the white light irradiation (100 mW/cm^2) for 0, 3, and 10 min and let stand in dark for 1 h. Then, HeLa cells were cultured with a medium containing 20 μg/mL preprocessed CP-NPs solution for 4 h and treated with medium containing 10 μM DHE solution for 30 min at 37 °C, followed by white light irradiation at 20 mW/cm^2 for 3 min. Afterward, the signal intensity was recorded by CLSM at excitation of 488 nm laser. Light alone and CP-NPs alone were stained like the above procedures. The emission filter was 580-650 nm.

PDT evaluation in vitro

Hela cells and 4T1 cells were respectively planted in 96-well plates with a density of 10000 cells per well and cultured one day. Prepared CP-NPs solution requires a self-degradable preprocessing under the white light irradiation (100 mW/cm^2) for 0, 3, and 10 min and let stand in dark for 1 h Then, the preprocessed CP-NPs (20 μg/mL) were added and incubated at 37 °C for 4 h in dark. After exposure to white light irradiation (20 mW/cm^2) for 3 min, the cells were further seeded at 37 °C to 4 h. CCK-8 assay was consistent with the description in Cytotoxicity

evaluation of CP-NPs.

Live/dead cell co-staining assay

HeLa cells and 4T1 cells were respectively incubated in a confocal imaging dish until the cell density reached 60-70% confluence. Prepared CP-NPs solution requires a self-degradable preprocessing under the white light irradiation (100 mW/cm^2) for 0, 3, and 10 min and let stand in dark for 1 h. Then, the preprocessed CP-NPs (20 μg/mL) were added and incubated at 37 °C for 4 h in dark. After exposure to white light irradiation (20 mW/cm^2) for 3 min, the cells were further seeded at 37 °C to 4 h. Afterward, the cells were stained with medium containing 2 μM Calcein-AM (AM) and 4 μM propidium iodide (PI) for 15 min. The cell imaging was recorded via a fluorescence microscope. PBS alone, light alone, and CP-NPs alone were stained like the above procedures.

In vivo imaging

All in vivo fluorescent images were recorded on the IVIS Lumina II In Vivo Imaging System (PerkinElmer). The nude mice bearing subcutaneous 4T1 tumor were given CP-NPs (200 μg/mL, 25μL/50mm^3 tumor) via intratumor injection. After injection of CP-NPs for 30 min, the tumor area of mice in the light group is irradiated by white light (200 mW/cm^2) for 20 min and the mice in no light group are untreated. Then fluorescent images were captured by the Lumina II at various time points. At 12 h of treatment with CP-NPs, the major organs and tumor tissues were collected for the imaging study.

PDT in vivo

All nude mice bearing subcutaneous 4T1 tumor mice were randomly divided into four groups (n = 5): “PBS”, “PBS + Light”, “CP-NPs”, and “CP-NPs + Light”. Thereinto, the intratumoral

injected dose of PBS and CP-NPs (200 μg/mL) is 25 μL/50 mm^3 tumor. After intratumoral injection, mice in "PBS + Light" and "CP-NPs + Light" groups were irradiated with a white light of 200 mW/cm^2 for 20 min, while other groups are not administered. During the treatment period, the weight and tumor size of mice in each group were measured once every 3 days for 18 days. Then the volume of tumors was calculated using the formula:

$V = (a \times b^2)/2$ where a is the length diameter and b is the width of the tumor.

On day 19, mice were sacrificed, and the tumor and major organs (heart, liver, spleen, lungs, and kidneys) were harvested. The collected tissues were immersed in 4% buffered paraformaldehyde, which was embedded into paraffin for further hematoxylin and eosin (H&E) staining. The histopathological changes were evaluated using an optical microscope.

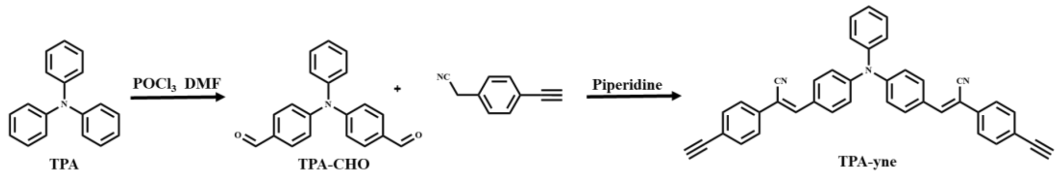

Figure S1. Synthetic routes of TPA-CHO and TPA-yne.

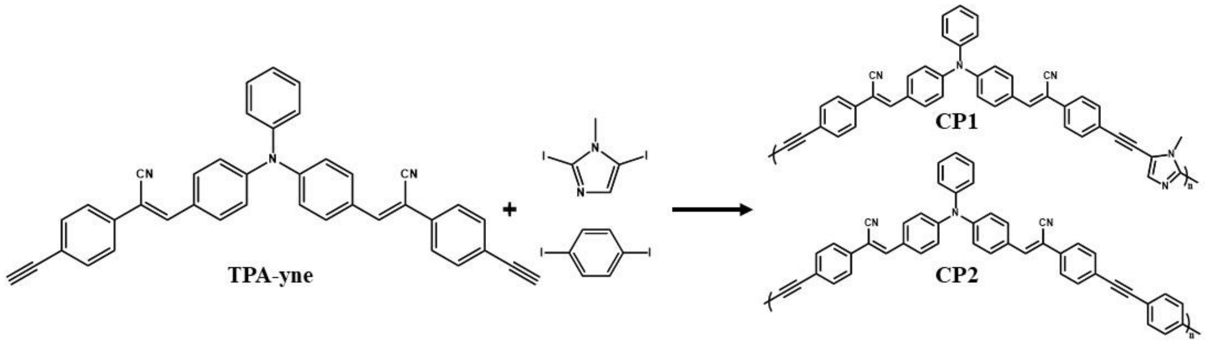

Figure S2. Synthetic routes of biodegradable CP1 and non-biodegradable CP2.

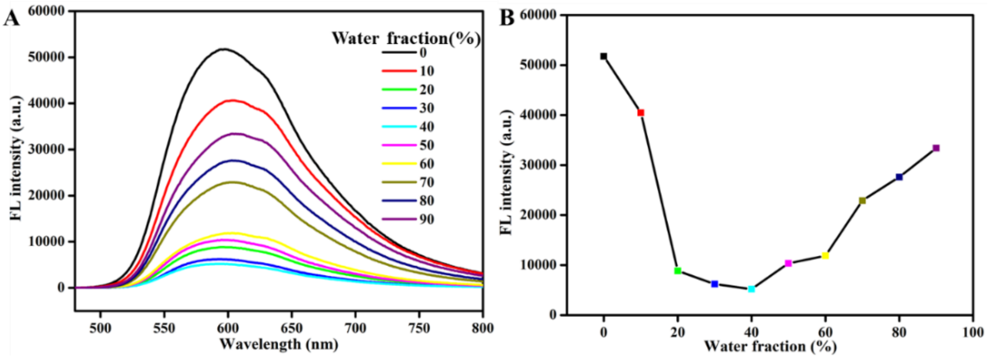

Figure S3. (A) Fluorescence spectra of CP1 in DMSO/water mixtures with different water fractions. (B) The relationship of fluorescent spectra of CP1 versus water fraction in the mixed solution.

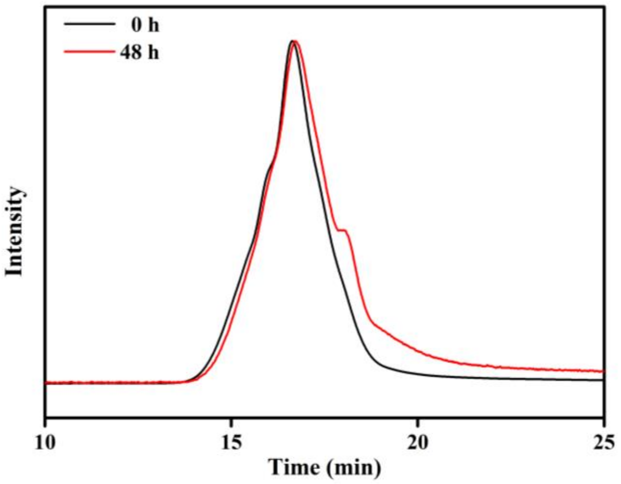

Figure S4. Normalized GPC results of the CP2 before and after exposure to H_2O_2 for 48 h.

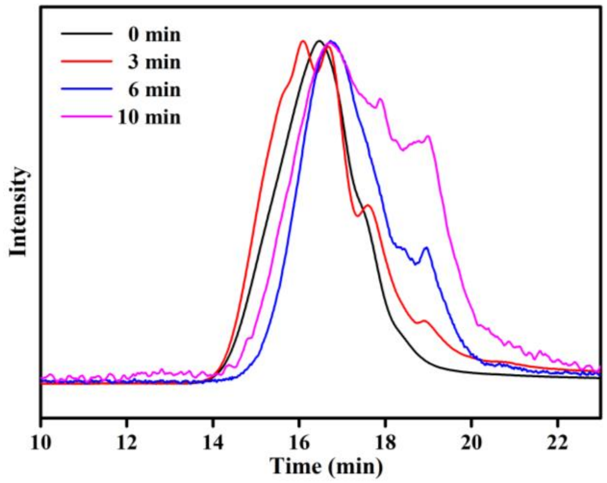

Figure S5. Normalized GPC results of CP1 with light irradiation for different time.

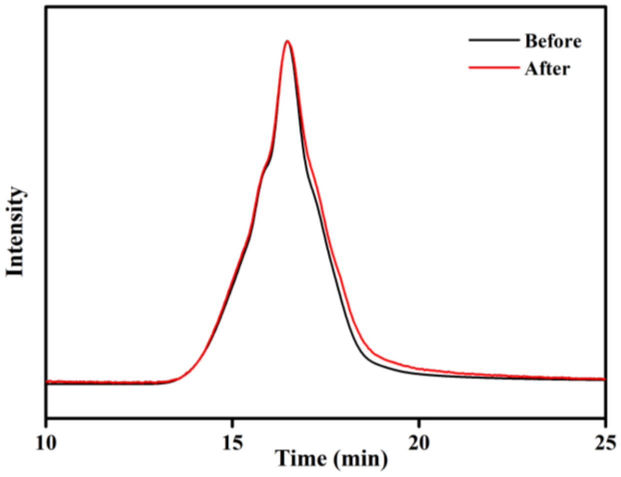

Figure S6. Normalized GPC results of CP2 before and after light irradiation for 10 min.

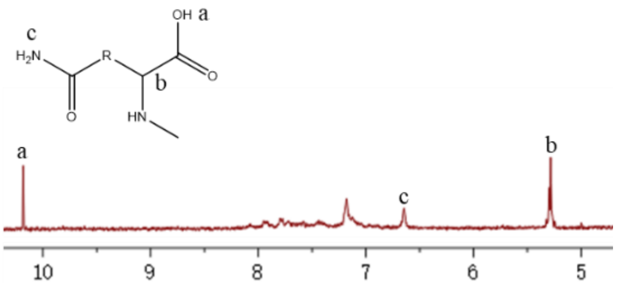

Figure S7. ^{1}H NMR spectrum of CP1 after light illumination in DMSO for 30 min.

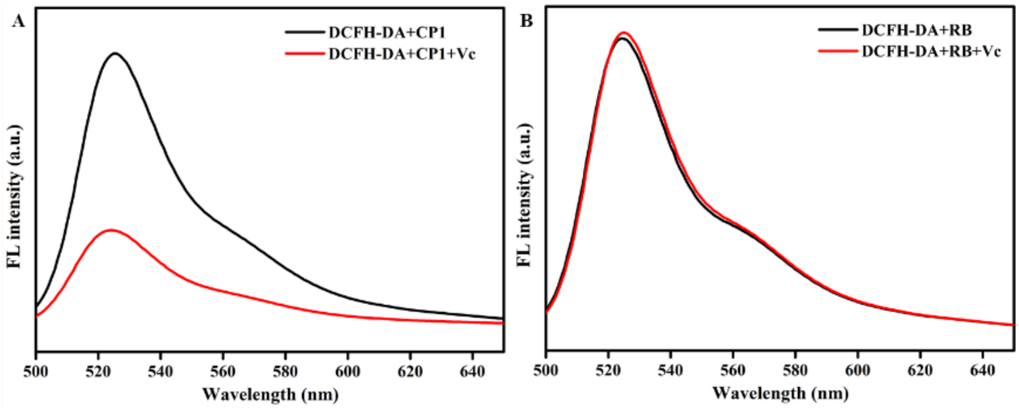

Figure S8. (A) Fluorescence spectra of DCFH-DA (50 μM) in the presence of CP1 (1 μg mL^{-1}) and Vc (10 μM) under irradiation for 3 min. (B) Fluorescence spectra of DCFH-DA (50 μM) in the presence of RB (1 μg mL^{-1}) and Vc (100 μM) under irradiation for 3 min.

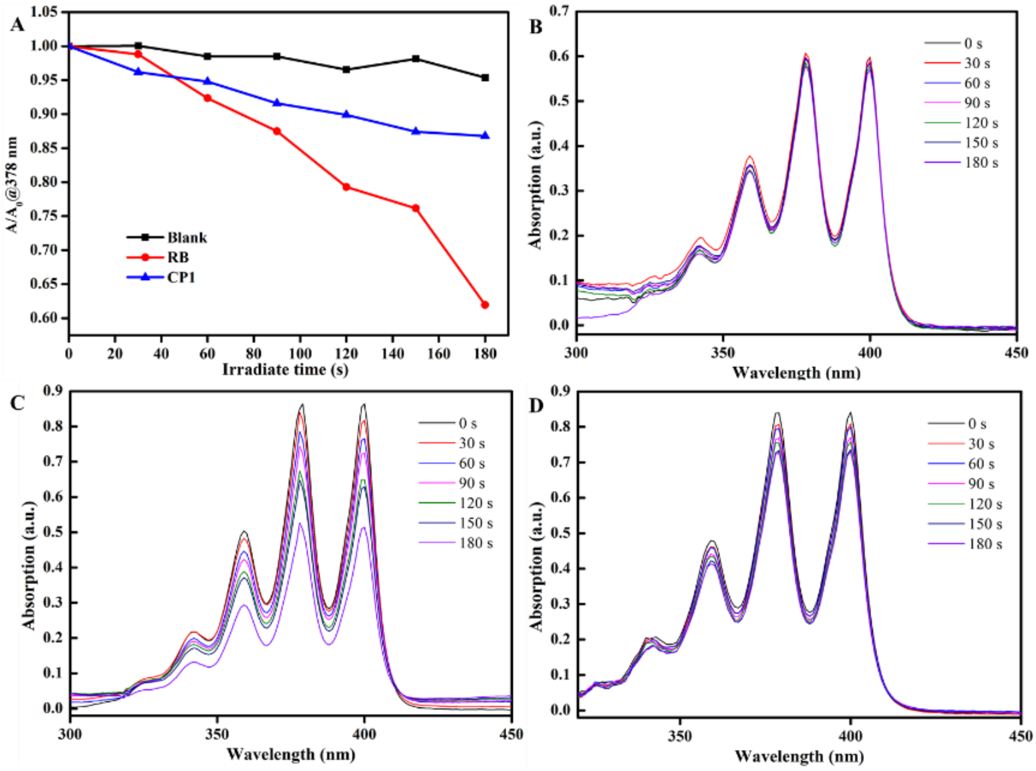

Figure S9. (A) Decomposition rates of ABDA (20 μM) solution containing RB (1 μg mL^{-1}) and CP1 (1 μg mL^{-1}). UV-vis spectra of ABDA in the presence of (B) Blank, (C) RB, (D) CP1.

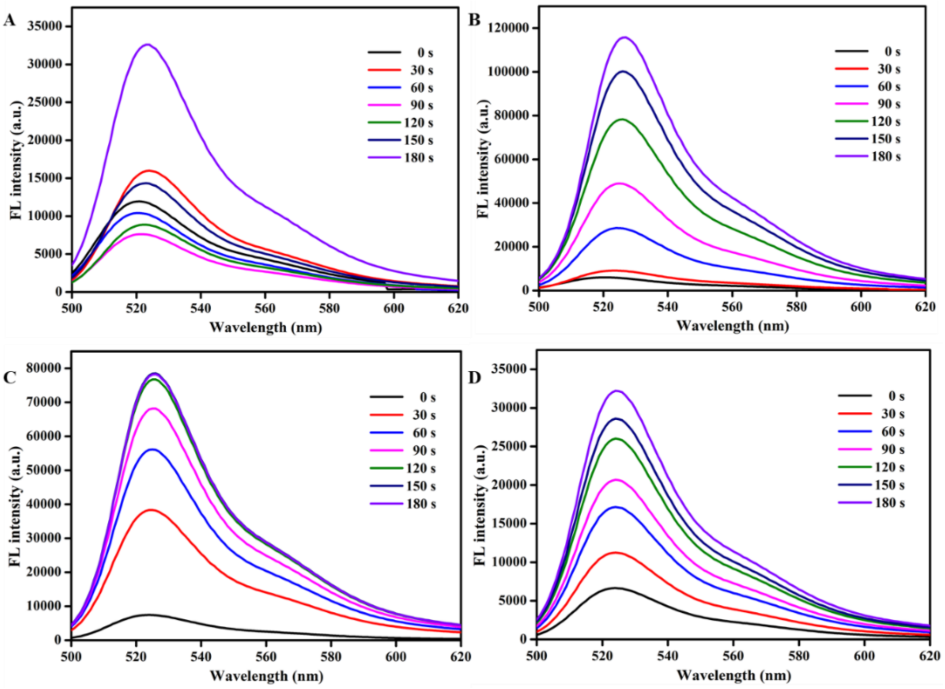

Figure S10. Fluorescence spectra of the DCFH-DA (50 μM) in the presence of CP1 (different pre-irradiation time) (1 μg mL^{-1}) under irradiation with different time (A) Blank, (B) untreated CP1, (C) 3 min pre-irradiation time CP1, (D) 10 min pre-irradiation time CP1.

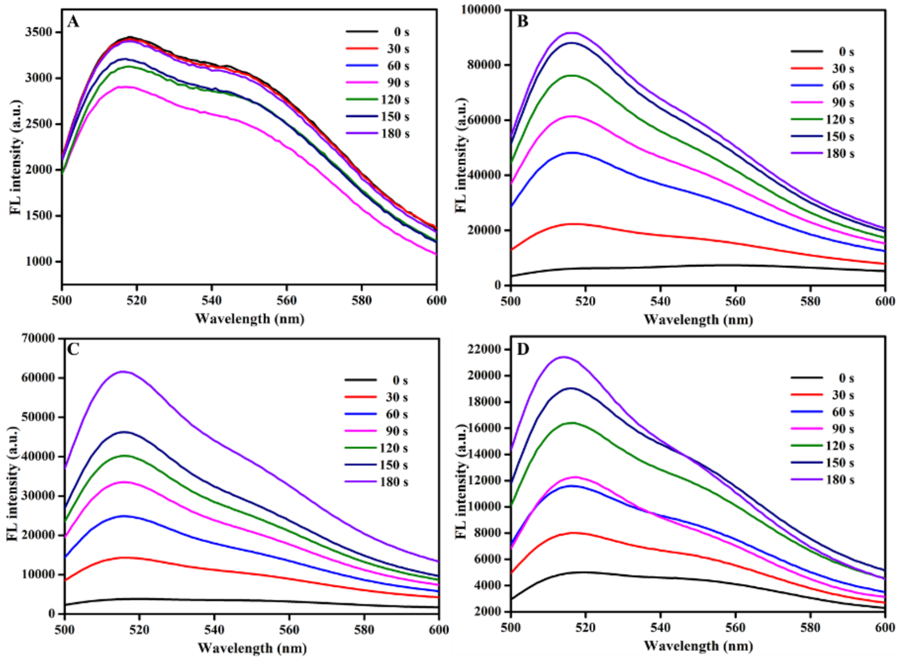

Figure S11. Fluorescence spectra of HPF (10 μM) in the presence of CP1 (different pre-

irradiation time) (1 μg mL^{-1}) under the irradiation with different time (A) Blank, (B) untreated CP1, (C) 3 min pre-irradiation time CP1, (D) 10 min pre-irradiation time CP1.

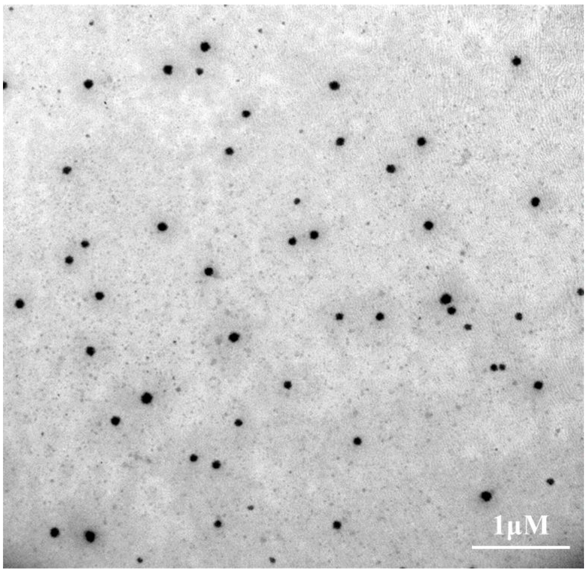

Figure S12. TEM imaging of CP-NPs. Scale bar, 1 μm.

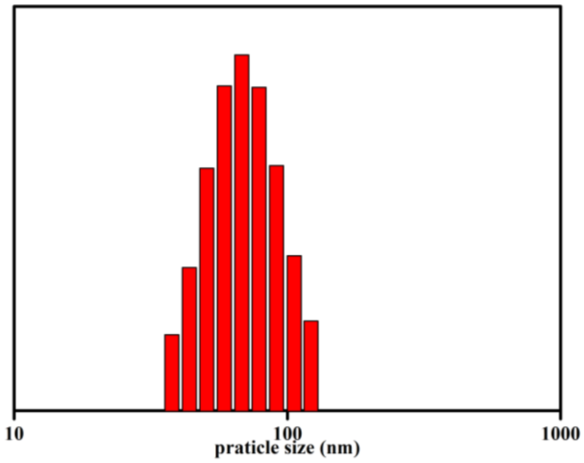

Figure S13. DLS data of CP-NPs.

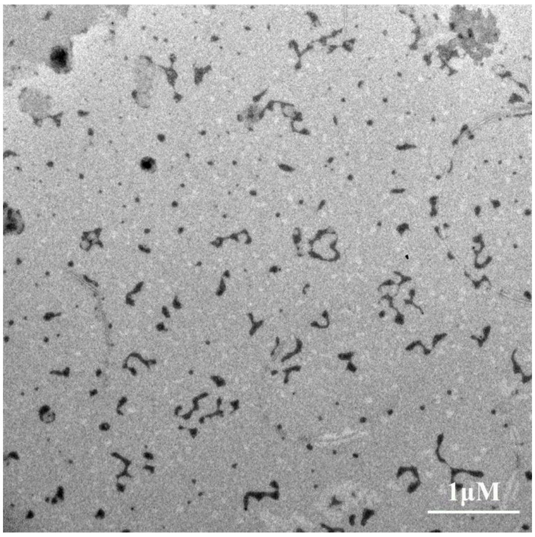

Figure S14. TEM imaging of CP-NPs after light. Scale bar, 1 μm.

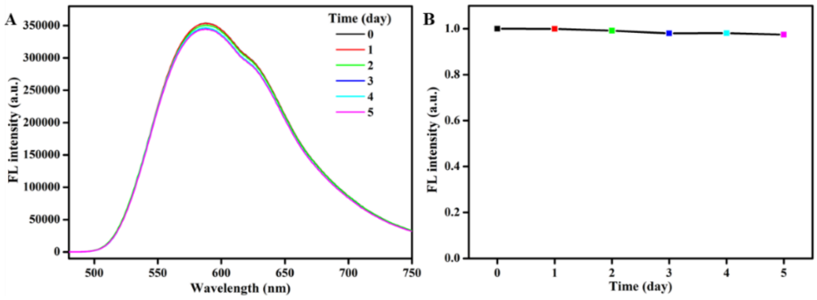

Figure S15. Fluorescence intensity of CP-NPs in PBS during 5 days.

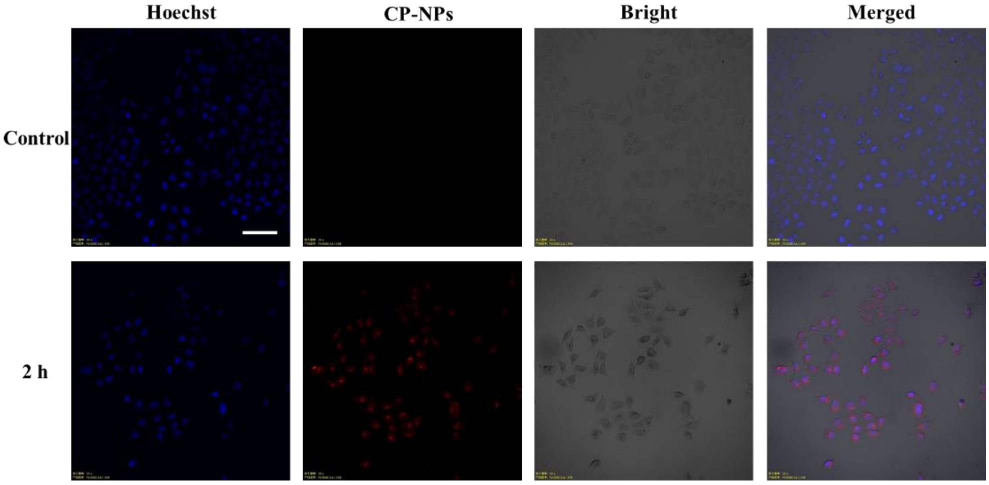

Figure S16. CLSM images of HeLa incubated with Hoechst33258 (1 μg mL^{-1}) and CP-NPs (20 μg mL^{-1}) for 2 h. Scale bar: 20 μm.

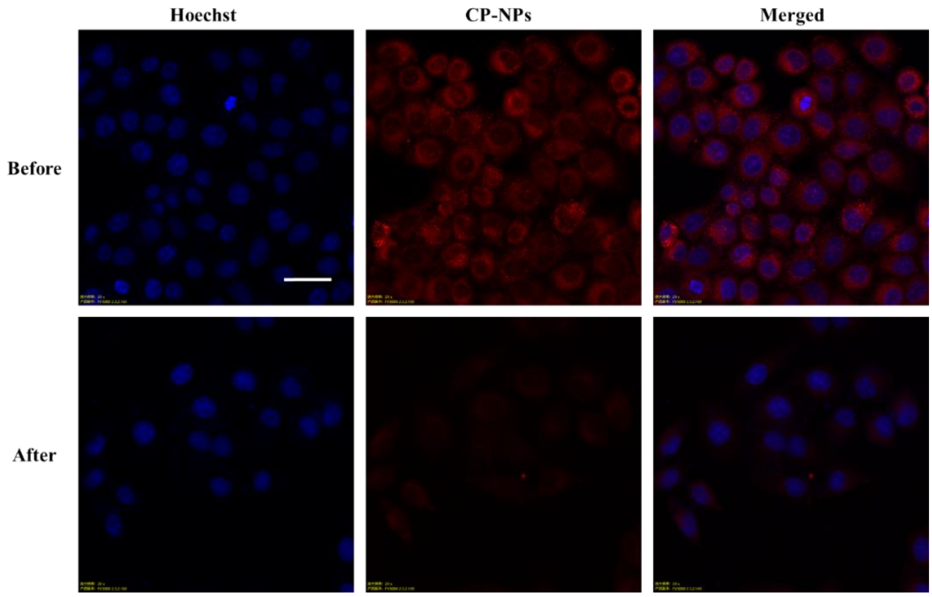

Figure S17. CLSM images of HeLa incubated with Hoechst33258 (1 μg mL^{-1}) and CP-NPs (20 μg mL^{-1}) before and after light irradiation for 10 min. Scale bar: 20 μm.

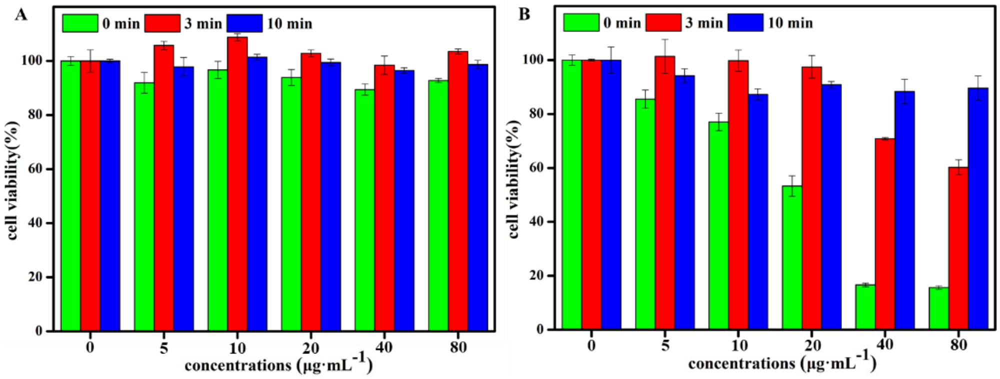

Figure S18. A) 4T1 cells viability with CP-NPs (different pre-irradiation time) under dark B) 4T1 cells viability with CP-NPs (different pre-irradiation time) upon white light irradiation.

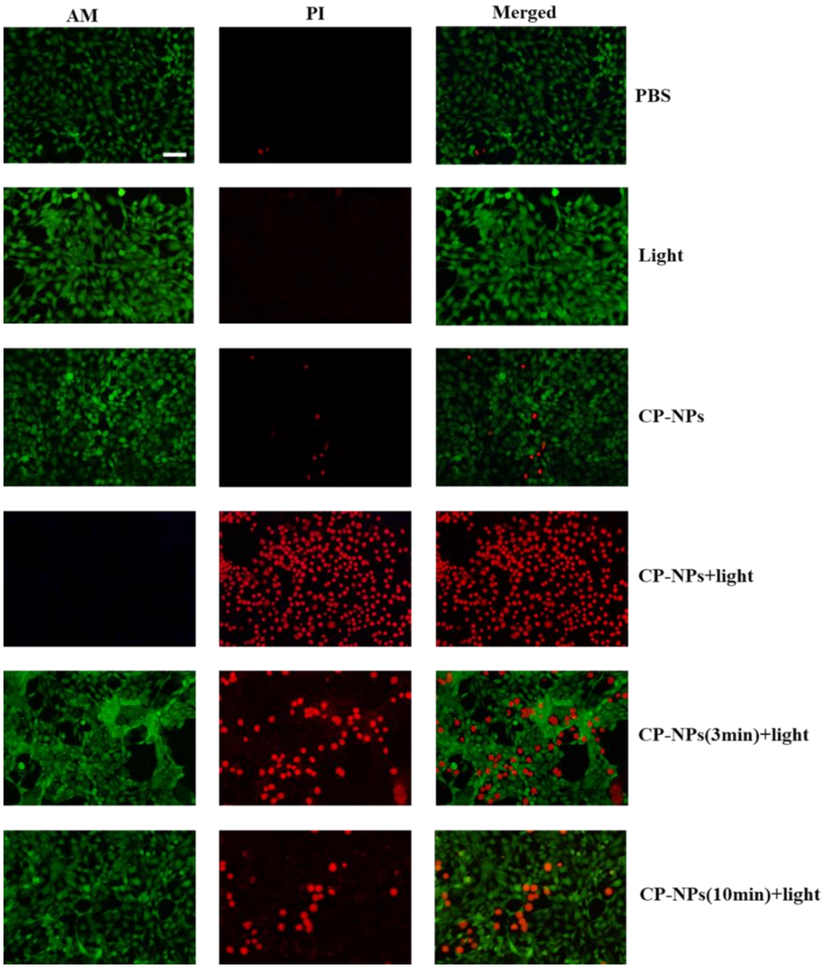

Figure S19. Cell images of PI/Calcein-AM (AM) co-stained 4T1 cells after CP-NPs (different pre-irradiation time) (20 μg mL^{-1}) or PBS incubation under different conditions. Scale bar: 50 μm.

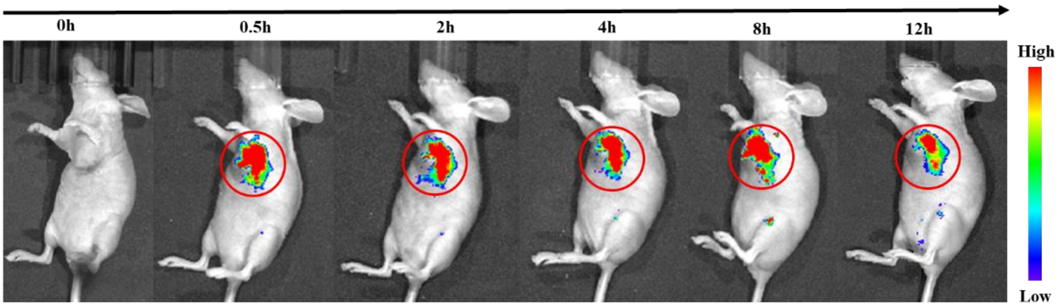

Figure S20. Fluorescence images of tumor-bearing nude mice after intratumoral injection of CP-NPs (200 μg mL-1) at different times.

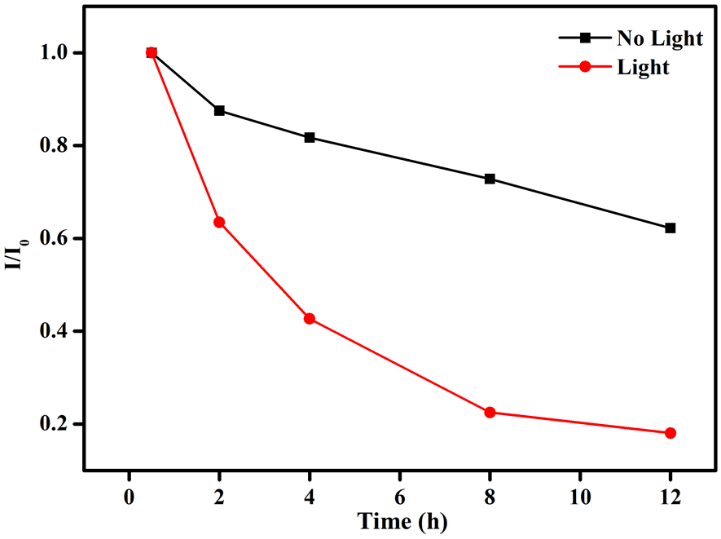

Figure S21. Variation in the intensity at different time points after intratumoral injection of CP-NPs. I_0 refers to the intensity of the tumor region at 0.5 h. The signal intensity of the tumor tissue was recorded on the regions enclosed by the red circles in Figure 5A and S20, respectively.

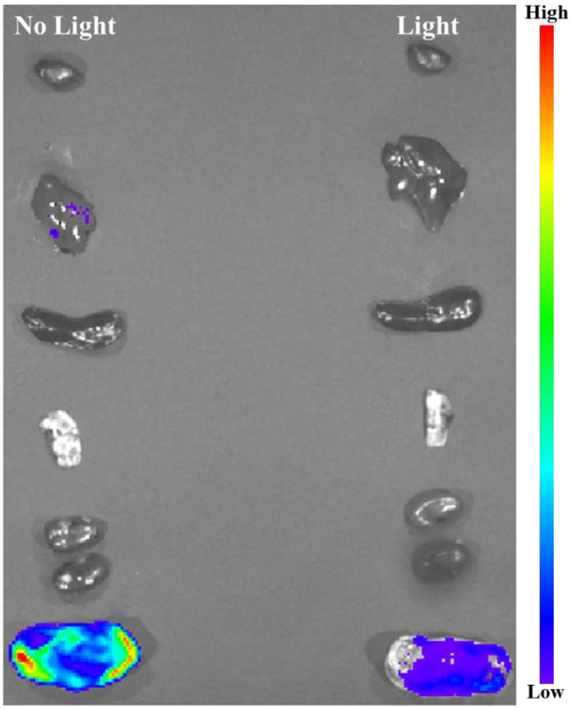

Figure S22. Ex vivo fluorescence imaging of Tumor and major organs after 12 h intratumoral injection of CP-NPs. The tumor area of mice in the light group is irradiated by white light (200 mW/cm^2) for 20 min and the mice in no light group are untreated.

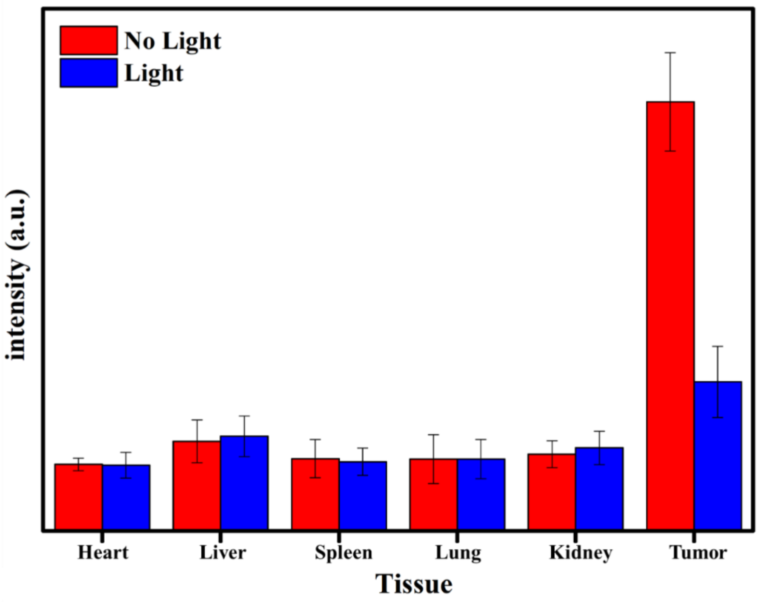

Figure S23. Histogram of the PL intensity of the tumor and organs (heart, liver, spleen, lung, and kidney) from light and no light group.

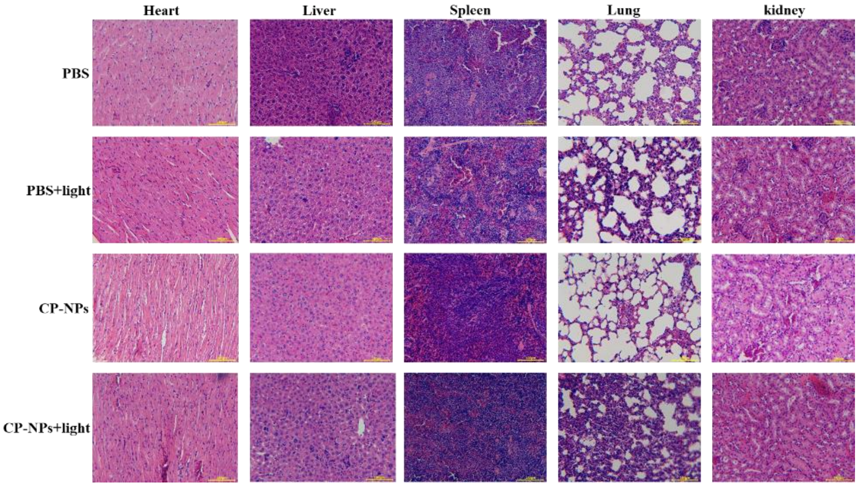

Figure S24. H&E staining of major organs of mice from different treatment groups after 7 days of treatment.

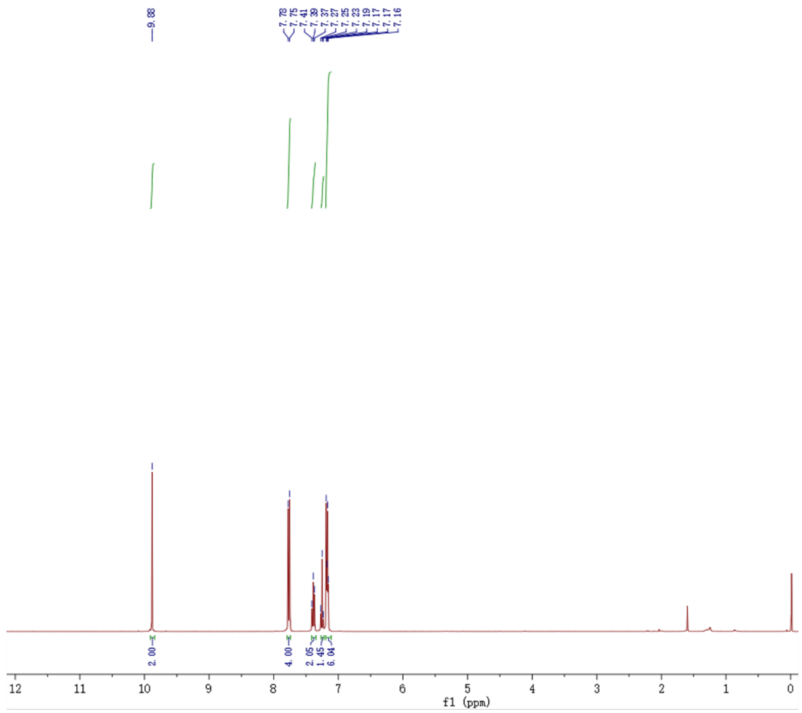

Figure S25. ^{1}H NMR spectrum of TPA-CHO.

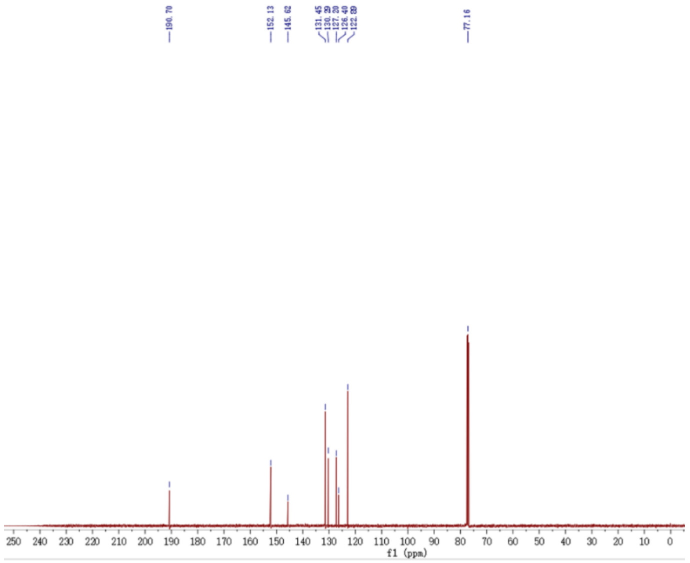

Figure S26. ^{13}C NMR spectrum of TPA-CHO.

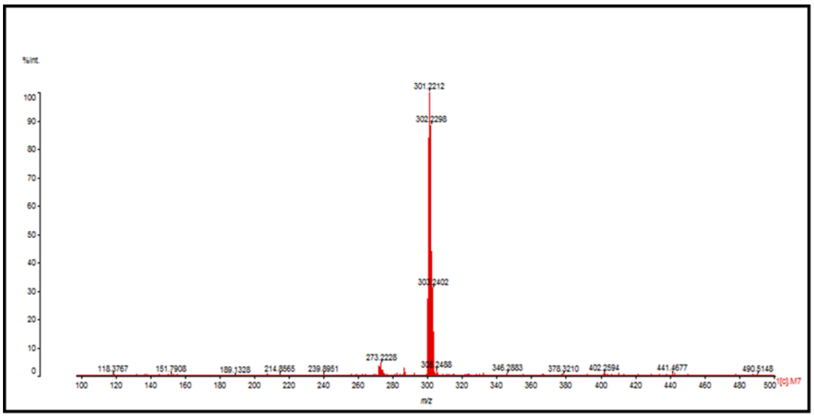

Figure S27. MALDI-TOF MS spectrum of TPA-CHO.

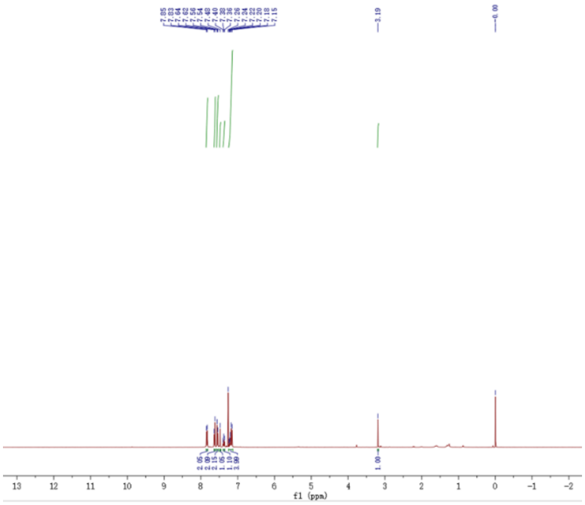

Figure S28. ^{1}H NMR spectrum of TPA-yne.

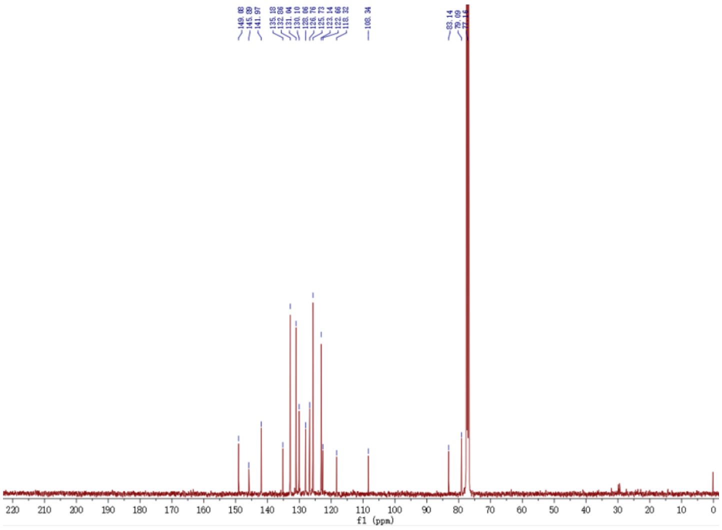

Figure S29. ^{13}C NMR spectrum of TPA-yne.

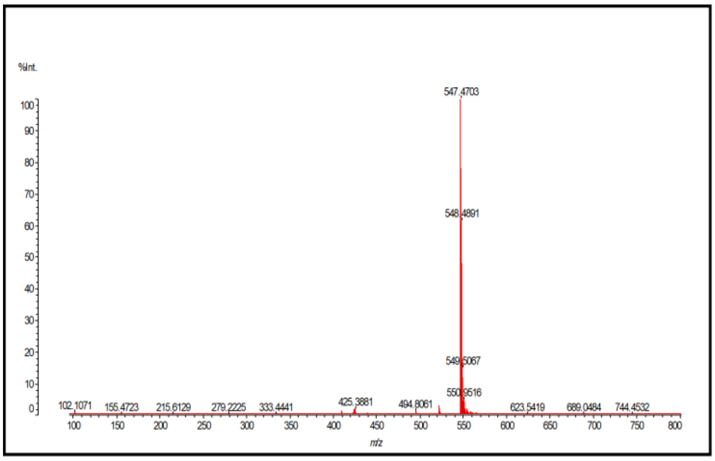

Figure S30. MALDI-TOF MS spectrum of TPA-yne.

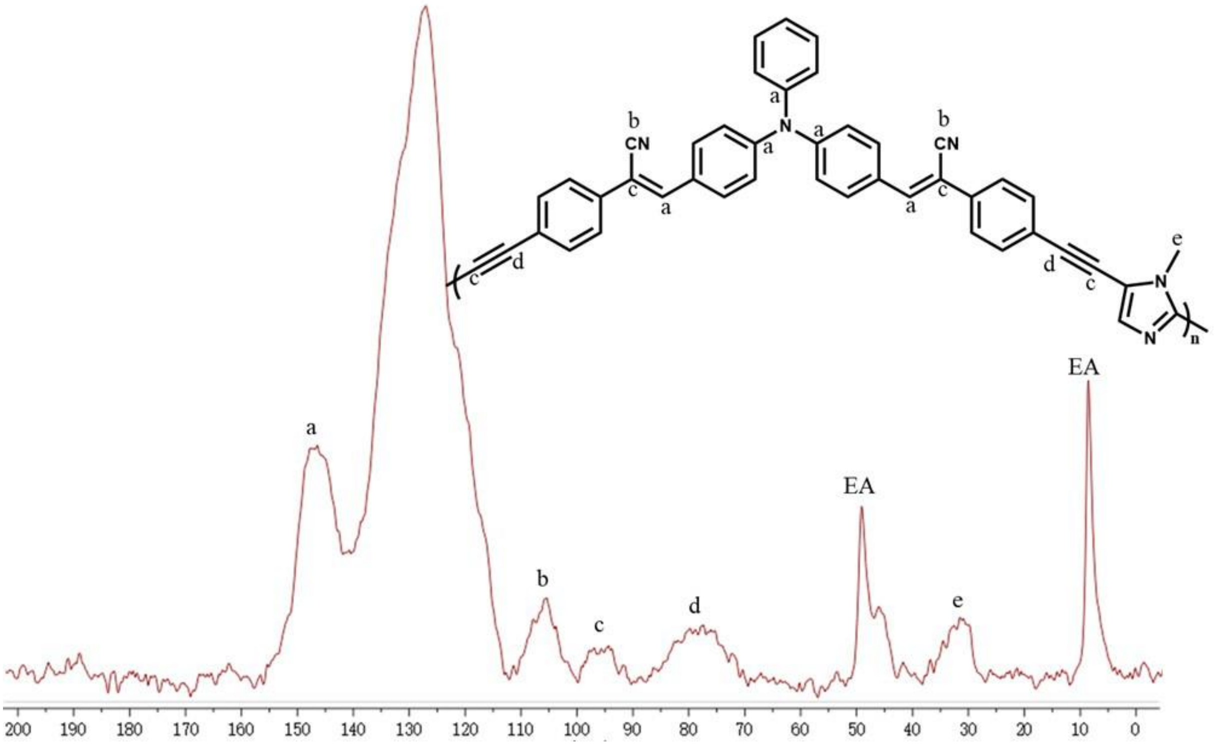

Figure S31. Solid ^{13}C NMR spectrum of CP1.

Table S1： GPC results of CP1 and CP2 exposure to H_2O_2 for different time.

Polymer (exposure of H_2O_2 time (h))	Mn	PDI
CP1（0）	7539	1.48463
CP1（24）	2809	2.40049
CP1（48）	692	2.57004
CP2（0）	6838	1.34250
CP2（48）	6057	1.37000

Table S2： GPC results of CP1 and CP2 with light irradiation for different time.

Polymer (light irradiation time (min))	Mn	PDI
CP1（0）	7493	1.38806
CP1（3）	4949	2.08255
CP1（6）	2755	2.35000
CP1（10）	1144	5.10200
CP2（0）	7262	1.45950
CP2（10）	6893	1.49979
